# Labor market participation and depression during the COVID-19 pandemic among young adults (18 to 30 years): A nationally representative study in France

**DOI:** 10.3389/fpubh.2022.904665

**Published:** 2022-10-24

**Authors:** Maria Melchior, Aline-Marie Florence, Camille Davisse-Paturet, Bruno Falissard, Cédric Galéra, Jean-Baptiste Hazo, Cécile Vuillermoz, Josiane Warszawski, Fallou Dione, Alexandra Rouquette

**Affiliations:** ^1^Sorbonne Université, INSERM, Institut Pierre Louis d'Epidémiologie et de Santé Publique (IPLESP), Equipe de Recherche en Epidémiologie Sociale (ERES), Faculté de Médecine St Antoine, Paris, France; ^2^Université Paris-Saclay, Inserm, Université Versailles Saint-Quentin (UVSQ), Centre d'Epidémiologie et de Santé Publique (CESP), Paris, France; ^3^AP-HP Paris-Saclay, Epidemiology and Public Health Department, Le Kremlin-Bicêtre, France; ^4^INSERM, Bordeaux Population Health Center, UMR1219, Bordeaux, France; ^5^French Ministry of Health-Statistical Service (Drees), Paris, France

**Keywords:** depression, epidemiology, young adult, COVID-19, labor market participation

## Abstract

**Objective:**

To examine the relationship between young adults' labor force participation and depression in the context of the COVID-19 pandemic.

**Design, setting, participants:**

Data come from the nationally-representative EPICOV cohort study set up in France, and were collected in 2020 and 2021 (3 waves of online or telephone interviews: 02/05/2020–12/06/2020; 26/10/2020–14/12/2020; 24/06/2021–09/08/2021) among 2,217 participants aged 18–30 years. Participants with prior mental health disorder (*n* = 50) were excluded from the statistical analyses.

**Results:**

Using Generalized Estimating Equation (GEE) models controlled for participants' socio-demographic and health characteristics and weighted to be nationally-representative, we found that compared to young adults who were employed, those who were studying or unemployed were significantly more likely to experience depression assessed using the PHQ-9 (multivariable ORs, respectively: OR: 1.29, 95% CI 1.05–1.60 and OR: 1.50, 1.13–1.99). Stratifying the analyses by age, we observed that unemployment was more strongly associated with depression among participants 25–30 years than among those who were 18–24 years (multivariable ORs, respectively, 1.78, 95% CI 1.17–2.71 and 1.41, 95% CI 0.96–2.09). Being out of the labor force was, to the contrary, more significantly associated with depression among participants 18–24 years (multivariable OR: 1.71, 95% CI 1.04–2.82, vs. 1.00, 95% CI 0.53–1.87 among participants 25–30 years). Stratifying the analyses by sex, we found no significant differences in the relationships between labor market characteristics and depression (compared to participants who were employed, multivariable ORs associated with being a student: men: 1.33, 95% CI 1.01–1.76; women: 1.19, 95% CI 0.85–1.67, multivariable ORs associated with being unemployed: men: 1.60, 95% CI 1.04–2.45; women: 1.47, 95% CI 1.01–2.15).

**Conclusions and relevance:**

Our study shows that in addition to students, young adults who are unemployed also experience elevated levels of depression in the context of the COVID-19 pandemic. These two groups should be the focus of specific attention in terms of prevention and mental health treatment. Supporting employment could also be a propitious way of reducing the burden of the COVID-19 pandemic on the mental health of young adults.

## Introduction

The COVID-19 pandemic has had a major impact on worldwide population mental health, still unresolved today (1). In particular, there is evidence of significant increases in the prevalence of major depression (up to ~27%) and anxiety disorders (up to ~25%), respectively, leading to an estimated 50 and 76 million new cases globally (1). In the general population, factors associated with a deterioration in mental health include female sex, loneliness, low income, and being a young adult (2).

Several studies have found that young adults who were university students experienced especially large increases in the prevalence of depression and anxiety disorders, with approximately a third suffering from either condition (3–5). However, many studies aiming to better understand the reasons why young people have been particularly impacted were conducted in China (3–5) where the spread of COVID-19 was limited after 2019 and accompanied by strict preventive measures, or in the United States (3) where to the contrary, the toll of the COVID-19 pandemic has been high and preventive measures such as distance teaching implemented for extended periods of time. Data from additional countries can help better understand the extent to which among young adults students are a high-risk group in terms of mental health during in the context of the current sanitary crisis.

This is particularly a question, given that young people are disproportionately likely to experience job instability and unemployment as entering the labor market without or with limited prior experience is challenging in many settings; both job instability and unemployment increased during the course of the COVID-19 pandemic and have been identified as risk factors of depression and anxiety (6–10). The association between unemployment and mental health has previously been observed and is considered to be partly bidirectional (11), indicating that it is essential to consider preexisting mental health difficulties in assessing changes in psychological distress during the course of the COVID-19 pandemic.

France is characterized by high levels of youth unemployment compared to other OECD countries: 18.5% among 15–24 year-olds in 2021, as compared with 11.5% on average (12). Additionally, a high proportion of youths are Not in Employment, Education or Training (NEET): 19% of 20–24 year-olds as compared to 15.5% on average (13). Youth unemployment appears to have increased to about 21% in 2020 before decreasing to about 18% by the end of 2021 (14), suggesting heightened pressure on young adults attempting to find employment and making France a relevant context to study relationships with mental health.

Moreover, France is among countries which were hit especially hard by the COVID-19 pandemic, particularly in 2020 and 2021. By March 2022, the COVID-19 pandemic has caused the death of over 140 000 persons (https://coronavirus.jhu.edu/region/france) and resulted in a strict national lockdown from March to May 2020, followed by two mitigated lockdowns from November 2020 to January 2021 and from April to June 2021. Between these periods, a curfew was implemented, and social and educational activities were restricted. According to a nationally representative survey of the French population conducted by the French Public Health Agency, the prevalence of symptoms of depression among 18–24 year-olds was estimated to be 16% in the Spring 2020 and 22% in January 2022, while, respectively, 33 and 43% suffered from symptoms of anxiety, that is double the rates observed prior to the COVID-19 pandemic (15). Similarly, a study conducted by the French Ministry of Health estimated, that in May and November 2020, respectively, 22 and 19 % of 15–24 years old experienced symptoms of depression while the prevalence prior to the COVID-19 pandemic was estimated to be 10% (16).

The aim of our study was to test the association between labor market participation and depression among young adults during the course of the COVID-19 pandemic. In particular, we compared young adults who were employed to those who were studying or unemployed, while accounting for sociodemographic and health factors potentially associated with depressive symptoms, including history of mental health problems prior to the COVID-19 pandemic.

## Materials and methods

### Study design

Data come from the EPICOV study, a French nationally-representative cohort designed to assess the main characteristics of the COVID-19 pandemic on the population of France as well as its impact in terms of sociodemographic and health outcomes (17). As previously described, participants (≥15 years of age, residing in mainland France or three out of five overseas territories) were randomly selected from the national tax database (FIDELI). FIDELI covers 96.4% of the population living in France, providing postal addresses for all individuals, and an e-mail address or telephone number for 83%. Sampling was stratified on two characteristics: residential area (“department”-equivalent to a county), and area-level poverty defined as 60% of the median household income per capita. This sampling frame was designed to ensure overrepresentation of less densely populated and more socioeconomically disadvantaged areas. Individuals living in residential care or prison were not invited to participate in the study.

All potential participants were contacted by post, e-mail and text messages (SMS), with up to seven reminders. Self-computer-assisted-web interviews (CAWI) were conducted with 80% of study participants. The remaining 20% were randomly assigned to CAWI or computer-assisted-telephone interviews (CATI).

The first wave of data collection took place in the spring 2020 (02/05/2020–12/06/2020) at the end of the first national COVID-19-related lockdown; the second in the fall 2020 (26/10/2020–14/12/2020), during the second national COVID-19-related lockdown; the third in the summer 2021 (24/06/2021–09/08/2021) after the end of the third national COVID-19-related lockdown.

### Study population

**Figure 1:**
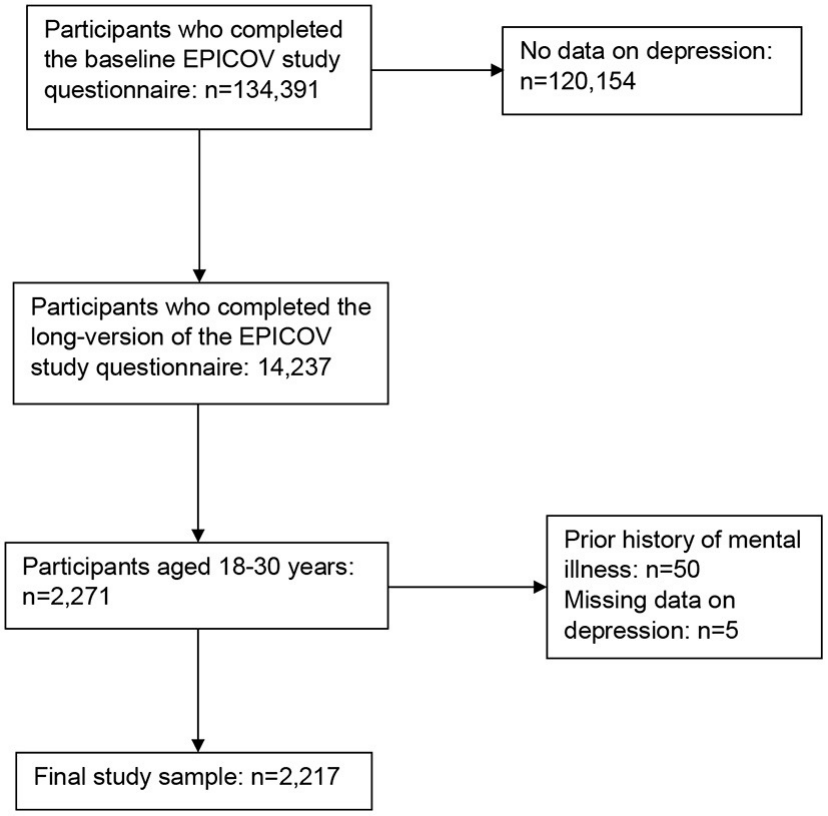
Flowchart showing the selection of EPICOV study participants aged 18–30 years and with available depression data.

Initially, 371,000 persons were randomly selected and 134,391 completed the first EPICOV study questionnaire (36.4% participation). Measures of mental health were included in an extended version of the study questionnaire completed by a random sample of 10% of study participants. As all EPICOV participants, this subsample was followed-up longitudinally (*n* = 14,237 persons in the first study wave, *n* = 12,519 in the second and *n* = 10,780 in the third study wave). Since our aim was to study determinants of depression among young adults, statistical analyses were restricted to participants aged 18–30 years who had at least one assessment of depression during the course of follow-up (*n* = 2,271 persons in the first, *n* = 1,966 in the second and *n* = 1,615 in the third study wave). Participants' aged 15–17 years at the time of the study were excluded because 95.57% were students, there was therefore no variability in labor force participation in this group. Additionally, to limit the influence of preexisting mental health difficulties on our results, we excluded study participants who had a prior history of depression or anxiety which limited their daily activities (*n* = 50) (Figure 1). In our study sample, 74.2% of participants completed the study questionnaire by CAWI and 25.8% by CATI.

### Measures

#### Study outcome

The primary outcome in our study was depression over the 2 weeks preceding the assessment, measured by the Patient Health Questionnaire−9 items (PHQ-9) score (18). Following previously published guidelines (18), participants with a score ≥ 10 were considered to have moderate to severe depression and were considered as cases.

#### Labor market participation

Participants' labor market participation was assessed at each study wave and treated as a time-varying exposure. We distinguished participants who reported that they were employed, studying, unemployed or out of the labor force (that is neither employed, unemployed, nor studying). Participants who reported studying and working were considered to be primarily students.

#### Covariates

Based on prior evidence, we selected several socio-demographic and health characteristics as possible confounding variables of the association between labor market participation and depression. Several characteristics were measured at study baseline: sex (female vs. male), age (25–30 vs. 18–24 years), urbanicity (rural area, <100,000 residents, ≥100,000 residents, Paris and suburbs), lone living (yes vs. no), having children (yes vs. no), and presence of a chronic disease (yes vs. no, the most frequent diseases reported were asthma-4.6%, physical disability-1.1%, gastro-intestinal disorder-0.9%, musculo-skeletal disorder-0.9%, respiratory disorder-0.6%, diabetes-0.5%). Other characteristics were studied as time-dependent variables: perceived financial situation (ascertained by the following question: “Do you consider your financial situation to be-comfortable, suitable, tight, difficult?”), romantic relationship (yes vs. no), outings in preceding week (<once, 2–5 times, >5 times), and experience of COVID-19 symptoms (yes vs. no, one or more symptoms among cough, fever, dyspnea, or ageusia, dysgeusia or anosmia).

### Statistical analyses

Reweighting and marginal calibrations were used to correct for non-participating bias and to take into account the effect of the means of data collection (Internet or telephone). To test the association between young adults' labor market participation and depression during the COVID-19 pandemic we proceeded as follows. First, associations were tested in bivariate Generalized Estimating Equations (GEE) (19) regression models weighted for study weights. Second, a multivariable GEE regression model was implemented including the study wave and all covariates associated with the study outcome with a *p*-value < 0.20 in bivariate models. GEE models are longitudinal regression models for clustered data which serve to estimate an average marginal (i.e., population) effect. In subsequent analyses, we repeated the analyses stratified on age (18–24 / 25–30 years), 25 being the age when youths in France cannot anymore be claimed as dependents by their parents. Additionally, we tested statistical interactions between participants' labor market participation and sex. Missing data on covariates (maximum 37%) were imputed using multiple imputations under the missing at random hypothesis, which we verified to hold (20). Data management and descriptive analyses were performed using R 4.0; weighted GEE models were implemented using SAS V9.4.

### Ethics approval

The EPICOV study received approval from an ethics committee (Comité de Protection des Personnes Sud Méditerranée III 2020- A01191-38) and from France's National Data Protection Agency (Commission Nationale Informatique et Libertés, CNIL, MLD/MFI/AR205138).

## Results

Across the three EPICOV study waves, the weighted prevalence of depression was, respectively, 17.1, 14.9, and 13.8%. As shown in Table 1, 45.0% of young adults participating in the study were employed, 40.6% were studying, 9.2% were unemployed and 5.2% were out of the labor force (that is not employed and not looking for employment, in general either because of health issues or parenting duties). 59.5% of participants were aged 18–24 years, and 49.2% were women. A majority resided in urban areas (39.2% in a town >100,000 residents–although this coefficient was not statistically significant and 21.3% in the Greater Paris area). Most participants had a suitable (38.4%) or tight (32.4%) financial situation at study baseline and 17.4% reported income below poverty level. 19.7% of participants reported living alone, 9.1% had children, 41.0% were in a relationship and at study baseline a majority reported leaving their home at least once a week (42.9% 2–5 times and 23.2% >5 times). In terms of health, 12.5% of participants reported having a chronic disease and, respectively, 18.0, 26.5, and 15.6% reported having experienced symptoms of COVID-19 in each of the three study waves.

**Table 1:** Descriptive characteristics of young adults (18–30 years) participating in the nationally representative EPICOV cohort study.

	**Unweighted**	**Depression**
	***N* (weighted %)**	**OR; 95% CI**
Labor market position: Employed	983 (45.0)	Ref
Student	928 (40.6)	1.76 [1.48–2.08]
Unemployed	194 (9.2)	2.05 [1.58–2.65]
Out of the labor force	112 (5.2)	1.77 [1.24–2.51]
**Socio-demographic characteristics**
Sex: Male	1,013 (50.8)	Ref
Female	1,204 (49.2)	1.59 [1.28–1.99]
Age (years): 18–24	1,347 (59.5)	Ref
25–30	870 (40.5)	0.64 [0.51–0.82]
Urbanicity: Rural area	376 (16.0)	Ref
<100,000 residents	530 (23.5)	1.38 [1.04–1.82]
≥100,000 residents	785 (39.2)	1.54 [1.20–1.98]
Paris and suburbs	393 (21.3)	1.59 [1.21–2.09]
Financial situation: Comfortable	399 (17.4)	Ref
Suitable	880 (38.4)	1.32 [1.02–1.69]
Tight	697 (32.4)	2.01 [1.56–2.59]
Difficult	234 (11.8)	3.90 [1.40–2.80]
Lone living: No	1,716 (80.4)	Ref
Yes	416 (19.7)	1.38 [1.15–1.65]
Has children: No	2,002 (90.9)	Ref
Yes	215 (9.1)	0.41 [0.27–0.64]
In a relationship: No	714 (41.0)	Ref
Yes	1,089 (59.0)	0.76 [0.63–0.92]
Outings in preceding week: <once	757 (33.9)	Ref
2–5 times	963 (42.9)	0.77 [0.64–0.94]
>5 times	497 (23.2)	0.54 [0.44–0.66]
**Health characteristics**
Chronic disease: No	1,932 (87.5)	Ref
Yes	285 (12.5)	1.66 [1.27–2.18]
COVID-19 symptoms: No	1,806 (82.0)	Ref
Yes	411 (18.0)	1.84 [1.54–2.20]

*France, 2020–2021, n* = *2,217, weighted % and bivariate Generalized Estimating Equations (GEE) regression models testing associations with depression*.

Table 1 also shows bivariate associations between labor market participation, covariates, and depression across follow-up.

In a multivariable weighted statistical model controlling simultaneously for all covariates (Table 2), compared to participants who were employed the odds of depression were elevated among those who were students (OR: 1.29, 95% CI 1.05–1.60) and among those who were unemployed (OR: 1.50, 95% CI 1.13–1.99). Other factors associated with depression were being a woman, being 25–30 years, living in an rural area, financial situation, lone living; outings in the preceding week, having a chronic disease and experience of COVID-19 symptoms.

**Table 2:** Association between labor market position and covariates and young people's (18–30 years) symptoms of depression.

		**OR**	**95% CI**
Labor market participation	Employed	Ref	
	Student	1.29	[1.05–1.60]
	Unemployed	1.50	[1.13–1.99]
	Out of the labor force	1.42	[0.98–2.08]
**Socio-demographic characteristics**
Study wave	1	Ref	
	2	0.90	[0.74–1.09]
	3	0.98	[0.78–1.24]
Sex	Male	Ref	
	Female	1.58	[1.34–1.86]
Age (years)	18–24	Ref	
	25–30	0.80	[0.65–0.98]
Urbanicity	Rural area	Ref	
	<100,000 residents	1.31	[0.97–1.74]
	≥100,000 residents	1.30	[1.01–1.69]
	Paris and suburbs	1.51	[1.13–2.01]
Financial situation	Comfortable	Ref	
	Suitable	1.38	[1.07–1.79]
	Tight	2.04	[1.57–2.64]
	Difficult	3.87	[2.93–5.33]
Lone living	No	Ref	
	Yes	1.31	[1.08–1.59]
Has children	No	Ref	
	Yes	0.42	[0.28–0.64]
In a relationship	No	Ref	
	Yes	0.85	[0.69–1.05]
Outings in preceding week	<once		
	2–5 times	0.80	[0.65–0.98]
	>5 times	0.62	[0.49–0.79]
**Health characteristics**
Chronic disease	No	Ref	
	Yes	1.74	[1.42–2.13]
COVID-19 symptoms	No	Ref	
	Yes	2.11	[1.77–2.51]

*Nationally representative EPICOV cohort study, France, 2020-2021, n* = *2,217, multivariable Generalized Estimating Equations (GEE) regression models, weighted OR, 95% CI*.

Stratifying the study population by age (Supplementary Table 1), we found that among participants aged 18–24 years, compared to those who were employed, the odds of depression were elevated among students (multivariable OR: 1.33, 95% CI 1.03–1.70) and participants out of the labor force (multivariable OR: 1.71, 95% CI 1.04–2.82). Among participants who were unemployed the likelihood of depression was elevated but the OR did not reach statistical significance (multivariable OR: 1.41, 95% CI 0.96–2.09). In the 25–30 years age group, compared to participants who were employed, we found elevated odds of depression among those who were unemployed (multivariable OR: 1.78, 95% CI 1.17–2.71).

Stratifying the study population by sex (Supplementary Table 2) we found no significant differences in the relationships between labor market participation and depression in men and women (compared to participants who were employed, multivariable ORs associated with being a student: men: 1.33, 95% CI 1.01–1.76; women: 1.19, 95% CI 0.85–1.67, multivariable ORs associated with being unemployed: men: 1.60, 95% CI 1.04–2.45; women: 1.47, 95% CI 1.01–2.15).

## Discussion

In a longitudinal nationally representative study of 18–30-year-olds, we found labor market participation to be associated with depression during the course of the COVID-19 pandemic in France. Young adults are a known high-risk group in terms of mental health; our study indicates that students and persons who are unemployed are especially vulnerable. As unemployment during and after the COVID-19 pandemic is likely to result mainly from mass lay-offs and reductions in work time, we believe that this is a propitious context to examine relations between unemployment and consequences on mental health. These results are robust to control for other characteristics associated with depression, highlighting the psychological needs of youths who are outside of the labor force and who should be the focus of psychological screening and intervention, particularly in the context of the COVID-19 pandemic.

### Mental health of students

Our results are in line with other studies which found elevated levels of depression and anxiety among university students (3–5, 21). First of all, students are vulnerable because of high levels of financial difficulties as well social isolation, which increased during the course of the COVID-19 pandemic (21, 22). Additionally, there is evidence that students have experienced specific issues during the course of the COVID-19 pandemic, such as worries related to their studies, difficulties finding motivation to study, missed learning opportunities (23) as well as increased conflicts with others and limited ability to study (24), which could fuel fears about their future and negative mood. There is suggestion that distance learning is especially complex for students from disadvantaged backgrounds and negatively contributes to their stress levels (25). Additionally, students are one of the groups–along with adolescents-in which use of screen-based media and devices increased particularly during the course of the COVID-19 pandemic (22). While this helped them stay in contact with friends and family and pursue coursework, inability to socialize and meet others probably hampered their ability to share worries and anxieties and seek social support.

### Mental health of young adults who are unemployed

Young adults are especially likely to experience job instability which we found to be associated with depression, consistently with other studies (6, 7, 26). Importantly, while persons who experience mental health difficulties may be especially likely to become unemployed (27), there is also evidence that the experience of unemployment predicts later symptoms of depression and anxiety (28). The COVID-19 pandemic, which had a major impact on employment irrespective of individuals' characteristics, is an interesting set-up to examine the relationship between unemployment and mental health, as pre-existing psychological difficulties are likely to play a lesser role than prior to the pandemic. Evidence showing that employment loss during the course of the sanitary crisis predicts psychological difficulties suggests that labor market characteristics could directly impact individuals' mental health (10). Mechanisms that could contribute to elevated levels of depression among young adults who are unemployed include financial difficulties, stress associated with uncertainty about labor market prospects, as well as social isolation, which can be an issue particularly for students living away from their family and close ones as well as for those who reduced social contacts with their friends (8, 10). One of the issues that remains unresolved at this point is whether depression which occurred among young adults during the COVID-19 pandemic will persist or predict other mental health difficulties over the longer-term.

### Study implications

The COVID-19 pandemic which has had massive effects on population mental health, highlighted the need to test and make available interventions preventing and treating persons who experience psychological distress and depression. Young adults have elevated risks of psychological distress, yet they are also one of the groups with lowest levels of access to mental healthcare at the population level (29). A recent umbrella review reported that psychosocial interventions as well as combined psychological and educational interventions lead by general practitioners are effective in preventing depression among youths (30). In addition, there is evidence that psychological counseling provided in the university context can effectively alleviate symptoms of depression among students (31). Moreover, mobile apps addressing signs of stress and psychological distress, which are effective and cost-effective, seem particularly well-suited to young adults who widely use screen-based media (32, 33). Mobile-based interventions and telemedicine may also be effective when addressing the mental health needs of young adults who are unemployed, although it may be more difficult to identify and target this group. Additionally, efforts aiming to favor young adults' employment also contribute to good mental health (34).

### Limitations and strengths

Our study has limitations which need to be acknowledged. First, the EPICOV study started in the Spring of 2020 and we have only retrospective data on participants' past history of mental health problems. Nevertheless, we were able to exclude from statistical analyses participants who had the most severe forms of psychopathology. It may be the case that some study participants did have less severe psychological difficulties prior to the study, which influenced their employment and health after the onset of the COVID-19 pandemic. While future studies should examine prospective changes in mental health among cohorts of young people studied prior to the COVID-19 pandemic, our results do not appear to be entirely explained by pre-existing severe mental health difficulties. Second, the EPICOV cohort suffered low participation rate (~36%), raising the issue of its representativeness. To address this, survey weights making the sample representative of the general population of France were calculated and included in all statistical analyses. Third, the EPICOV cohort study is also characterized by attrition, with participants belonging to socioeconomically disadvantaged groups least likely to participate over the long term (17). This lead us to conduct repeated measures analyses, making it possible to maximize the use of information provided by study participants and limit possible biases due to attrition. Moreover, our statistical analyses are weighted to render the study sample representative of young adults in France. Fourth, in the EPICOV cohort, depression was ascertained with the PHQ-9 questionnaire, which is self-reported. While clinical diagnoses may yield a more accurate picture of severe depression, the PHQ-9 has satisfactory validity vs. clinical diagnosis (18) and we specifically studied a level of symptoms consistent with moderate to severe depression.

Our study also has strengths which we would like to highlight. First, EPICOV is a nationally-representative sample of the population residing in France and the results are generalizable to the whole country (15). Second, to our knowledge, ours is one of few investigations in the context of the COVID-19 pandemic to directly compare young people in different labor market positions, including those who are out of employment and who are generally more difficult to include in health cohort studies. Third, EPICOV participants were followed-up from the Spring 2020 to the Fall 2021, making it possible to study participants' mental health through different phases of the COVID-19 pandemic. Fourth, the use of online as well as telephone interviews and multiple call-backs helped include participants more reluctant to take part in health surveys in the study sample.

### Conclusions

Young adults experience high levels of depression in the context of the COVID-19 pandemic, students and the unemployed being at higher risk than young adults who are employed. Our findings imply that young adults who are in training or looking for work should be the focus of attention in terms of prevention and mental health treatment. In particular, efforts should be deployed to improve access to effective psychosocial interventions and mental health treatment for persons who are unemployed. Moreover, strategies that enhance young people's chances of finding employment could have benefits in terms of mental health.

## The EPICOV study group

JW and Nathalie Bajos (joint principal investigators), Guillaume Bagein, François Beck, Emilie Counil, Florence Jusot, Nathalie Lydié, Claude Martin, Laurence Meyer, Philippe Raynaud, Alexandra Rouquette, Ariane Pailhé, Delphine Rahib, Patrick Sillard, Alexis Spire.

## Data availability statement

The raw data supporting the conclusions of this article will be made available by the authors, without undue reservation.

## Ethics statement

The studies involving human participants were reviewed and approved by Comité de Protection des Personnes Sud Méditerranée III 2020- A01191-38 and France's National Data Protection Agency (Commission Nationale Informatique et Libertés, CNIL, MLD/MFI/AR205138). The patients/participants provided their written informed consent to participate in this study.

## Author contributions

The study was designed and conceptualized by MM and AR. A-MF and FD conducted statistical analyses. All authors contributed to data interpretation and validated the final version of the manuscript.

## Funding

This research was supported by research grants from Inserm (Institut National de la Santé et de la Recherche Médicale) and the French Ministry for Research, Drees (Direction de la Recherche, des Etudes, de l'Evaluation et des Statistiques), the French Ministry for Health, and the Région Ile de France. Dr. Bajos has received funding from the European Research Council (ERC) under the European Union's Horizon 2020 research and innovation program [Grant No. (856478)]. This project has also received funding from the European Union's Horizon 2020 research and innovation program under Grant No 101016167, ORCHESTRA (Connecting European Cohorts to Increase Common and Effective Response to SARS-CoV-2 Pandemic).

## Conflict of interest

The authors declare that the research was conducted in the absence of any commercial or financial relationships that could be construed as a potential conflict of interest.

## Publisher's note

All claims expressed in this article are solely those of the authors and do not necessarily represent those of their affiliated organizations, or those of the publisher, the editors and the reviewers. Any product that may be evaluated in this article, or claim that may be made by its manufacturer, is not guaranteed or endorsed by the publisher.
